# Differential effect on acute pulmonary perfusion according to mechanical support in acute myocardial infarction cardiogenic shock: an acute animal model

**DOI:** 10.1186/s40635-025-00809-w

**Published:** 2025-10-22

**Authors:** Stéphane Manzo-Silberman, Bart Meyns, Guillaume Lebreton

**Affiliations:** 1 Institut de Cardiologie, Hôpital de la Pitié-Salpêtrière, APHP, Sorbonne Université, 47-83 Bd de l’Hôpital, 75651 Paris Cedex 13, France; 2 Department of Cardiac Surgery, University Hospital UZ Leuven, Louvain, Belgium; 3 Service de Chirurgie Cardio-Vasculaire, Hôpital de la Pitié-Salpêtrière, APHP, Sorbonne Université, 47-83 Bd de l’Hôpital, 75651 Paris Cedex 13, France

**To the Editor**

Recent advances in mechanical circulatory support (MCS) devices have provided new avenues for the management of cardiogenic shock (CS). Veno-arterial extracorporeal membrane oxygenation (VA-ECMO) and micro-axial flow pumps (mAFP), such as the Impella device, are two technologies that have been increasingly utilized in clinical practice [1]. VA-ECMO, by providing complete cardiopulmonary support, helps in maintaining systemic perfusion and oxygenation. However, concerns have been raised about its potential to increase left ventricular afterload, which might exacerbate myocardial ischemia [2]. In contrast, mAFP reduce myocardial oxygen demand, potentially offering superior myocardial protection. As left ventricle function and coronary flow have long monopolized the spotlight, pulmonary perfusion in CS has been underappreciated so far. To better understand the nuanced impacts of these devices in the early phase of cardiogenic shock, we employed a hyperacute (treatment < 3h) animal model of ischemic cardiogenic shock to compare the influence of these circulatory supports on early hemodynamic changes, direct effect on myocardial perfusion, consumption of oxygen, and on pulmonary hemodynamic parameters.

Methodology: In 10 juvenile female Swifter sheep, after ketamine infusion anesthesia was induced by isoflurane via mask, under mechanical ventilation myocardial infarction was induced by temporary ligature of the first dominant diagonal branch of the LAD following a left lateral thoracotomy. To allow assessment of the pulmonary flow, a flow probe was attached to the main pulmonary artery. Over a period of 90 min (T1), the animals developed cardiogenic shock and subsequently were randomized to mechanical hemodynamic support either by peripheral VA-ECMO or mAFP. Reperfusion was performed after 60 min of assistance (T2) and the final assessment 120 min later (T3). Details of the models have been published previously, along with the main results regarding left hemodynamic parameters with cardiac output, pressure–volume loops, and myocardial energy consumption analyses [3]. All experiments were approved by the Leuven ethical board (approval ECD No. 193/2014) (supplemental material).

Results: Infarct size as percentage of area at risk was similar between groups (VA-ECMO: 28.25 ± 21.5% vs. mAFP: 24.03 ± 6.41%; *p* = 0.681). Following the onset of mechanical circulatory support (T1), both groups were stabilized to a sustainable mean arterial pressure (Table 1). During ischemia, left ventricle end-diastolic pressure (LVEDP) had increased in all animals. Interestingly, while on MCS prior to reperfusion, LVEDP decreased considerably in the mAFP group as compared to the VA-ECMO group; at the same time, left ventricular volume was not reduced (Fig. 1S). Differences were seen in pulmonary flow, where VA-ECMO led to a drop in pulmonary flow over time, whereas mAFP preserved pulmonary circulation (Fig. 1). Reperfusion did not translate into a change in pulmonary flow irrespective of the device.

**Table 1:** Hemodynamic parameters over time

MAP (mmHg)	T0	T1	T2	T3
VA-ECMO	52.2 ± 18.71	41.2 ± 18.78	55.2 ± 18.46	41 ± 11.14
mAFP	61.6 ± 7.64	59 ± 22.37	56.2 ± 14.02	51.6 ± 9.81
CVP (mmHg)				
VA-ECMO	10 ± 6.12	9 ± 2.92	9.6 ± 7.27	8.2 ± 5.07
mAFP	6.8 ± 6.69	7 ± 2.45	5.8 ± 2.28	5.6 ± 2.41
LVEDP (mmHg)				
VA-ECMO	8.09 ± 5.62	15.09 ± 5.3	19.29 ± 11.91	11.4 ± 7.4
mAFP	7.14 ± 1.8	12.31 ± 6.62	7.37 ± 2.06	6.53 ± 3.18
HR (bpm)				
VA-ECMO	95 ± 28.37	74.6 ± 26.96	55 ± 8.16	99.4 ± 94.28
mAFP	97 ± 17.71	91.4 ± 21.17	84.2 ± 9.52	86.25 ± 20.69

All values are presented as mean ± standard deviation

*CVP* central venous pressure, *HR* heart rate, *LVEDP* left ventricle end-diastolic pressure, *MAP* mean arterial pressure

**Fig. 1:**
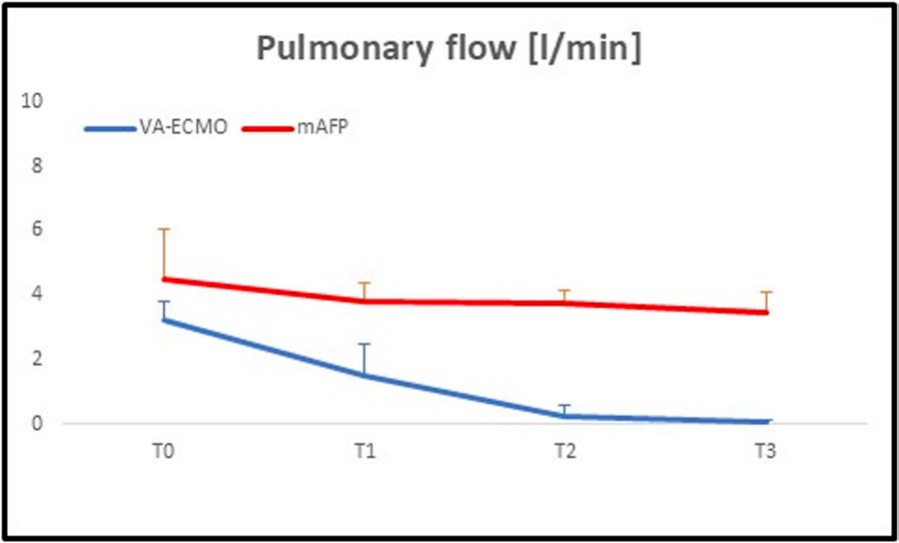
Pulmonary flow

The present model provided original data on pulmonary flow, one of the surrogates of right ventricle function, and how the different support strategies impacted it. Depletion of pulmonary blood flow by VA-ECMO, despite enabling systemic circulatory support, leads to increased pulmonary vascular resistance over time. Our model only allows us to assess the effect in the acute phase, but VA-ECMO can affect pulmonary function through various pathophysiological mechanisms [4]*.* In contrast, the mAFP, by unloading the left ventricle through provision of forward flow, preserved pulmonary circulation. The differences in clinical outcome cannot be fully explained by the hyperacute effects of the distinct mechanical support systems on left-sided hemodynamic parameters alone. In cardiogenic shock, differential effect on pulmonary flow may impact global circulation and influence outcome [5–8]. Further investigation into the differential effects on pulmonary vascular resistance and right ventricular afterload between these devices is warranted.

## Supplementary Information

Supplementary Material 1. Figure 1S: Left ventricular end-diastolic pressure (A) and end-diastolic volume (B) over time.

## Data Availability

The materials and methods have been already published [3]. The raw data supporting the findings of this study will be made available by the authors, without undue reservation, to any qualified researcher.
