# Supplementary material for: Differential effect on acute pulmonary perfusion according to mechanical support in acute myocardial infarction cardiogenic shock: an acute animal model
Source: Intensive Care Med Exp. 2025 Oct 22;13:104. doi: 10.1186/s40635-025-00809-w (PMC12545937; doi:10.1186/s40635-025-00809-w)
Supplement: Supplementary file 1 — Supplementary Material 1. Figure 1S: Left ventricular end-diastolic pressure (A) and end-diastolic volume (B) over time. [file 40635_2025_809_MOESM1_ESM.docx]

**Supplemental material**

**Material and Methods**

All experiments were approved by the Leuven ethical board (approval ECD No. 193/2014). Experiments were carried out in 10 juvenile female Swifter sheep, weighing 42-56 kg (48 ± 4 kg). After administration of ketamine (10-20 mg/kg body weight, intravenous), anesthesia was induced with isoflurane (5%, Iso-vet 1000mg/g, Piramal Critical Care, Rouboslaan 32, 2252 TR, Voorschoten, Nederland) via face mask. Prior to intubation with an endotracheal tube (8 mm, Coviden™, Shiley™), animals were preoxygenated for 3-5 minutes. Anesthesia was maintained with isoflurane (2-2.5%) and mechanical ventilation (11-14/min; tidal volume 7ml/kg body weight; positive end-expiratory pressure at 2 cm water after chest opening) during the surgery. A peripheral venous line (16 GA, BD Insyte™ Autoguard™ BC Winged) and central venous catheter (7 Fr, 3 lumens, 20 cm, Arrow) were placed in the left saphenous and jugular vein, respectively. Peripheral arterial pressure was measured invasively (20 GA, BD Insyte™ Autoguard™ BC Winged) in an ear artery via a fluid-filled line connected to a pressure transducer (Maquet GmbH, Rastatt, Germany). A large 23 Fr gastric tube was placed in the stomach to prevent ruminal distension. Before every procedure, a temperature probe (9Fr, YSI 400, OP&S) was established in the rectum. Pulse oximetry was secured to the tail or the tongue. Five leads were placed on the animal for electrocardiogram monitoring, with one on each limb and one on the abdomen. The animal was placed on the operating table in a right lateral recumbent position and surgically scrubbed and draped to expose the left thoracic region.

Prophylactic antibiotics (penicillin 40000 IU/kg and gentamycin 6.6 mg/kg) were given intravascular before first skin incision. For rhythm stabilization, animals were primed with 300mg amiodarone and 0.5mg/kg/h lidocaine was continuously administered. An f_i_O_2_ of 0.25 was maintained, animals were kept at normocapnic levels (including necessary reductions of ventilation while on VA-ECMO). During the entire experiment, phenylephrine was the only vasoactive/inotropic medication allowed to maintain a mean arterial pressure above 50mmHg. In case of ventricular fibrillation, external defibrillation was allowed to regain a stable rhythm.

Following the complete instrumentation of the animal, baseline values (T0) were taken, and the protocol was carried out as demonstrated in figure 1. Following a left lateral thoracotomy, the pericardium was opened and both access to the left coronary system and the pulmonary trunc was prepared. To allow assessment of the pulmonary flow, a flow probe (MA20PAU, Transonic Europe B.V., Elsloo, Netherland) was attached to the main pulmonary artery. Size of the flow probe liner was determined by visual inspection of the pulmonary trunc (Disposable Ultrafit Liner Chronic (closed) liner 18 mm or 20mm, Transonic Europe B.V., Elsloo, Netherland). Following preparation of the left main stem and LAD, the first dominant diagonal was sought and proximally ligated. Following 90 min of ischemia, either VA-ECMO cannulae (jugular 21Fr venous, femoral 17Fr arterial) or a transfemoral micro-axial flow pump (femoral 14Fr, Impella CP, Abiomed Europe GmbH, Aachen, Germany) were inserted and mechanical support was initiated at 3.5L/min (VA-ECMO) or maximum achievable unloading (mAFP). After 60 minutes on support, reperfusion was initiated by release of the ligature that staid in situ. Following 120 minutes of reperfusion, to assess infarct area at risk and infarcted myocardium the ligature was re-closed followed by an intracoronary injection of 50ml Evans blue (2%) per coronary vessel before the animal was euthanized by thiopental. The heart was then excised, thoroughly washed, and cut into equal slices of 1cm thickness. Area at risk was determined as the unstained area of the left ventricle prior to a 5min staining with 1%TTC at 37°C to determine the infarction zone.

Throughout the experiment, heart rate, pressures in aorta, left ventricle, vena cava, pulmonary artery, and device flows as indicated by the device-specific consoles were continuously recorded. Pressure volume loops to calculate left ventricular end systolic and end diastolic pressure and volume, stroke volume, native cardiac output and total pressure volume area were assessed at baseline (T0), after 90 min of ischemia prior to initiation of MCS (T1), prior to reperfusion following 60 min of MCS (T2) and after 2 hours of reperfusion while on continued support (T3). Blood samples were drawn at the same time points to measure arterial pH and lactate, carbon dioxide, creatine kinase (CK), troponin, hematocrit, hemoglobin, fibrinogen and plasma free hemoglobin in addition to central venous oxygen saturation.

**Figure 1S: Left ventricular end-diastolic pressure (A) & end-diastolic volume (B) over time**

***
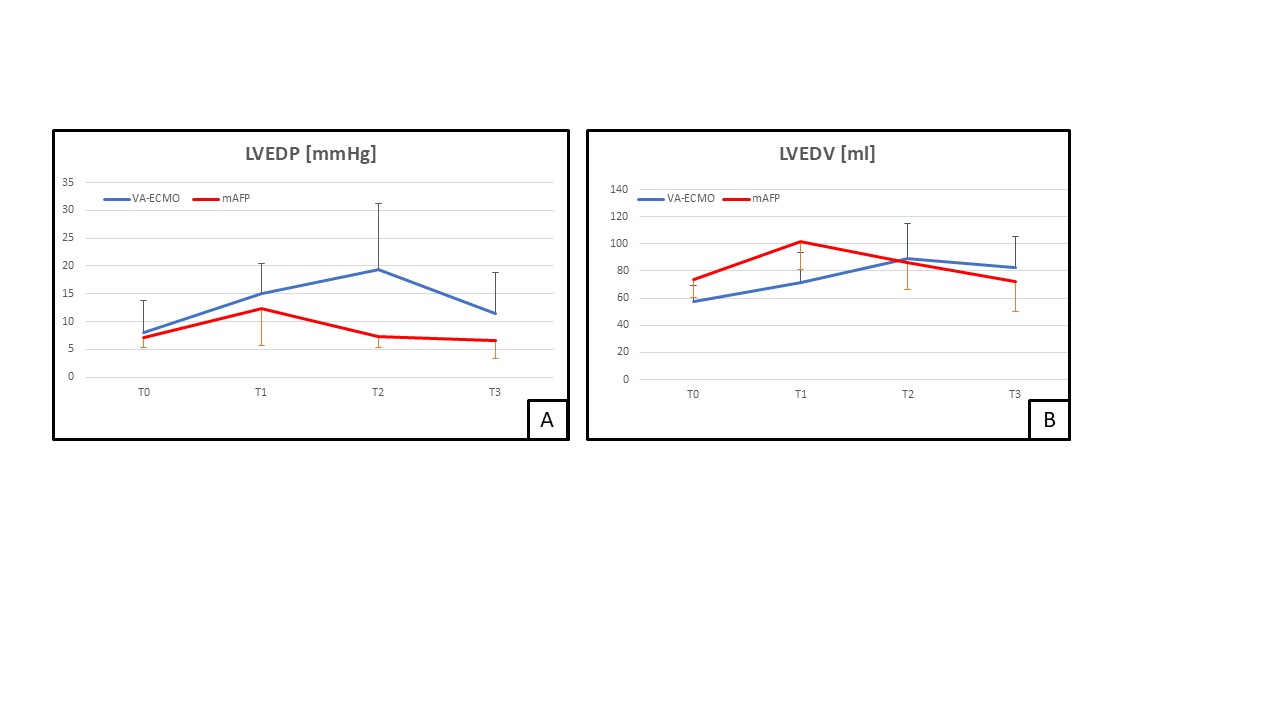
***
